# Posterior interosseous nerve syndrome caused by a ganglion cyst and its surgical release with intraoperative neurophysiological monitoring: A case report

**DOI:** 10.1097/MD.0000000000024702

**Published:** 2021-02-26

**Authors:** Dougho Park, Dong Young Kim, Yoon Sik Eom, Sang-Eok Lee, Seung Bum Chae

**Affiliations:** a Department of Rehabilitation Medicine; b Department of Orthopedic Surgery, Spine and Joint Center, Pohang Stroke and Spine Hospital, Pohang; c Department of Orthopedic Surgery, Daegu Catholic University Hospital, Daegu, Republic of Korea.

**Keywords:** electromyography, ganglion cyst, intraoperative neurophysiological monitoring, nerve conduction, posterior interosseous nerve syndrome

## Abstract

**Rationale:**

Intraoperative neurophysiological monitoring (IONM) has been utilized not only for the rapid detection of neural insults during surgeries, but also to verify the neurophysiological integrity of nerve lesions in the surgical field.

**Patient concerns:**

A 32-year-old woman presented with a wrist and finger drop that had lasted about 3 months.

**Diagnoses:**

The result of the initial electrodiagnostic test was consistent with posterior interosseous nerve (PIN) syndrome. Ultrasonography and magnetic resonance imaging of the proximal forearm showed a cystic mass at the anterolateral aspect of the radial head, which was diagnosed as a ganglion cyst.

**Interventions:**

Surgical release of the ganglion cyst with IONM was performed. During the surgery, we induced nerve action potentials and compound motor action potentials across the ganglion cyst, which demonstrated neural continuity.

**Outcomes:**

Three months after the surgery, the patient showed partial recovery of wrist and finger extensor muscle power. An electrodiagnostic test conducted 3 months after the surgery showed reinnervation potentials in PIN-innervated muscles.

**Lessons:**

IONM during peripheral nerve surgeries can support surgical decisions and confirm the location and degree of nerve damage.

## 1 Introduction

Posterior interosseous nerve (PIN) syndrome refers to an entrapment neuropathy caused by compression of the PIN, a deep branch of the radial nerve, just distal to the elbow.^[1]^ PIN syndrome is rare and can be caused by trauma, mass lesions, inflammation, and repetitive stress.^[2]^ A few case reports have indicated ganglion cysts as the cause of radial nerve compression.^[3–5]^

Intraoperative neurophysiological monitoring (IONM) is a useful tool that can determine the degree of nerve damage and accurately localize the lesion during peripheral nerve surgery.^[6]^ A representative IONM modality utilized in peripheral nerve surgery is nerve action potential (NAP), which can assess the neurophysiological continuity of the nerve.^[7]^ Inching tests, consisting in the induction of compound motor action potential (CMAP) through direct nerve stimulation and muscle recording, can also be utilized.^[8]^

This case report presents a rare case of a patient who underwent surgical treatment with IONM for PIN syndrome caused by a ganglion cyst.

## 2 Case presentation

**Figure 1:**
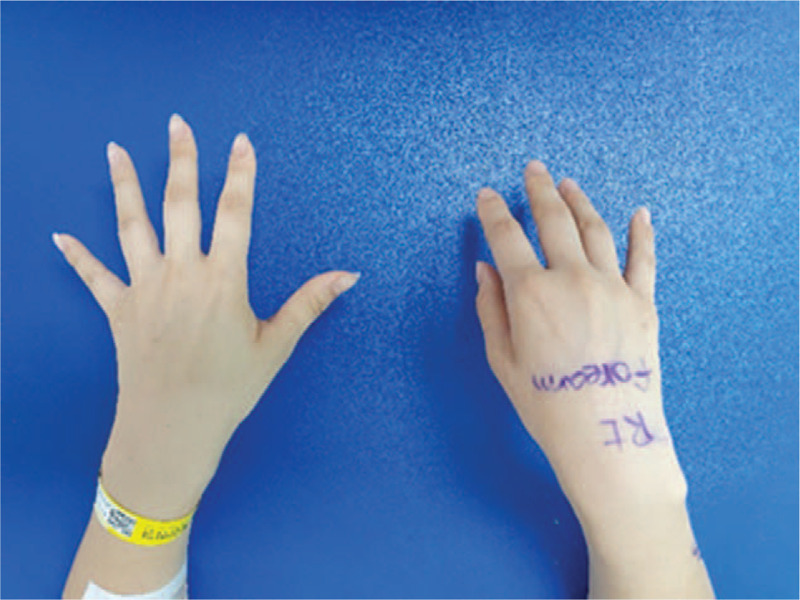
Preoperative gross appearance of the patient's bilateral wrist and hand showing wrist and finger drop with muscle atrophy on the right side.

A 32-year-old woman visited our hospital with a right side wrist and finger drop that had lasted about 3 months prior to the visit (Fig. 1). The patient had been treated conservatively in a local private clinic after the onset of symptoms. However, the symptoms did not improve. Consequently, she was referred to our center. She had type 1 diabetes in her medical history, was using an insulin pump, and her blood sugar level was under stable management. The patient was fully informed about this study and provided informed consent. This case report was also reviewed and approved by the Institutional Review Board of our institution (approval No. PSSH0475-202010-HR-011-01).

### 2.1 Clinical assessment and electrodiagnostic evaluation

Preoperative muscle strength was measured as grade 2+ at the wrist extensor and grade 1 at the finger and thumb extensors, according to the modified Medical Research Council scale (mMRC). Sensory symptoms were not observed.

An electrophysiological test was carried out to identify exact etiology and disease severity. The nerve conduction study (NCS) showed that the radial CMAP amplitudes recorded in the right extensor indicis proprius (EIP) and extensor digitorum communis (EDC) muscles were less than 50% of those on the left side. Superficial radial sensory nerve action potential showed normal findings. Electromyography (EMG) showed denervation potentials in the EDC, abductor pollicis longus (APL), extensor pollicis longus (EPL), and EIP muscles. Therefore, we diagnosed the patient with radial nerve palsy at the proximal forearm level, and specifically PIN syndrome.

### 2.2 Imaging Findings

**Figure 2:**
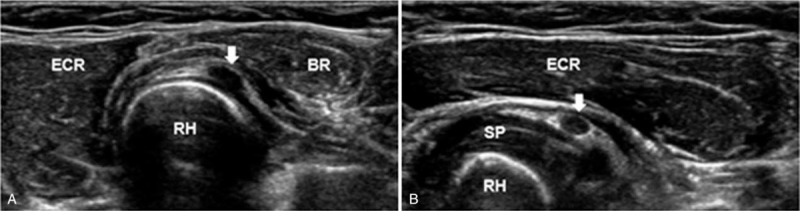
Axial A, and longitudinal B, ultrasonographic findings of the patient's right proximal forearm, showing a hypo-echoic, well demarcated ganglion cyst (arrows) located on the anterolateral side of the radial head. BR = brachioradialis, ECR = extensor carpi radialis, RH = radial head, SP = supinator.

**Figure 3:**
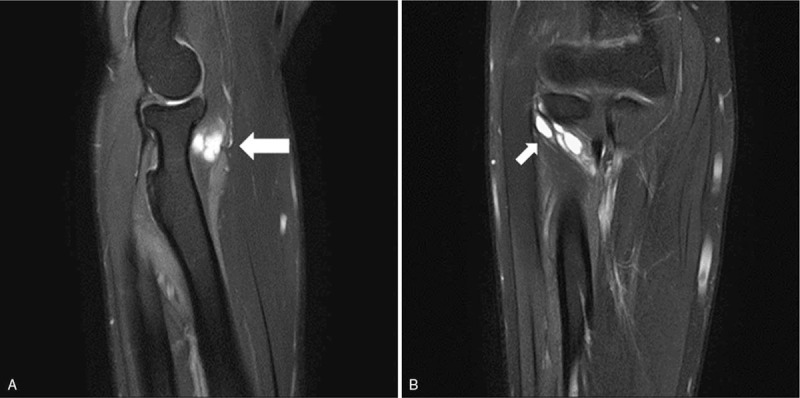
Preoperative magnetic resonance imaging (MRI) findings. A multilobulated cystic lesion anterior to the radial head (arrows) was shown in the sagittal (A) and coronal (B) views of MRI.

An ultrasonographic study conducted just after the electrodiagnostic test revealed a 2-cm hypoechoic and clearly bounded cystic mass on the anterior side of the radial head (Fig. 2). For a more detailed assessment, we conducted magnetic resonance imaging (MRI) to identify and localize the lesion more accurately. MRI revealed a multilobulated cystic mass at the anterolateral aspect of the radial head with high signal intensity on the T2 image (Fig. 3). We interpreted this mass as a ganglion cyst. We also considered the mass to have caused paralysis by compressing the PIN, and decided to proceed with surgical treatment. The patient underwent cervical spine MRI at the same time to rule out any accompanying root lesion or myelopathy.

### 2.3 Surgical procedures and intraoperative neurophysiological monitoring

**Figure 4:**
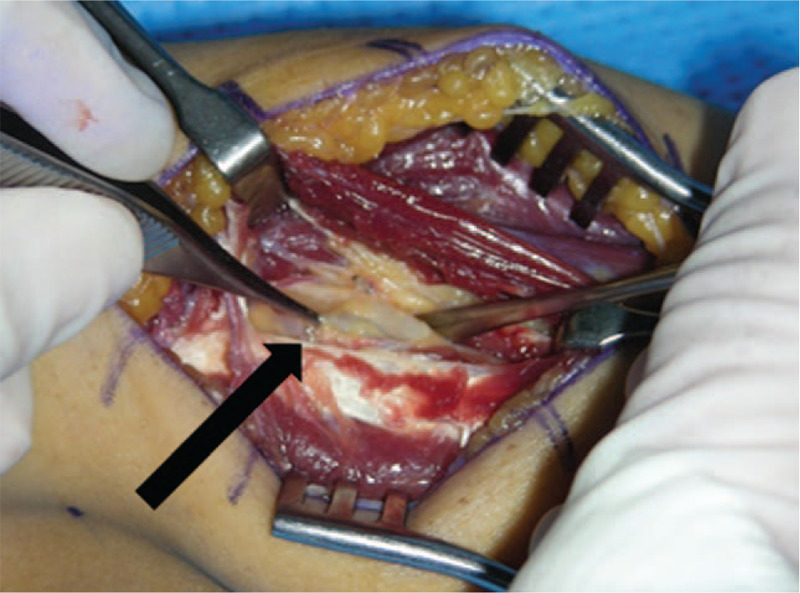
Gross appearance of the surgical field, showing the posterior interosseous nerve located on the anterolateral aspect of the elbow, and compressed by the ganglion cyst (arrow).

For the surgical approach, a curved skin incision was made along the lateral border of the brachioradialis muscle, and dissection was performed on the intermuscular plane of the brachioradialis and extensor carpi radialis longus muscles. After dissecting the superficial muscle layer, we checked the ganglion cyst and found that the PIN was compressed between the ganglion cyst and the supinator muscle. Then, the ganglion cyst was removed, and nerve atrophy was seen not only in the area where the ganglion cyst was located, but also near the arcade of Frohse (Fig. 4).

During the surgery, MEP monitoring was performed on the EDC, EIP, EPL, and abductor digiti minimi muscles. Free-run EMG was also monitored throughout the surgery. After PIN dissection, direct nerve stimulation was performed to obtain NAP and CMAP to confirm the state of the neural lesion and its integrity.

**Figure 5:**
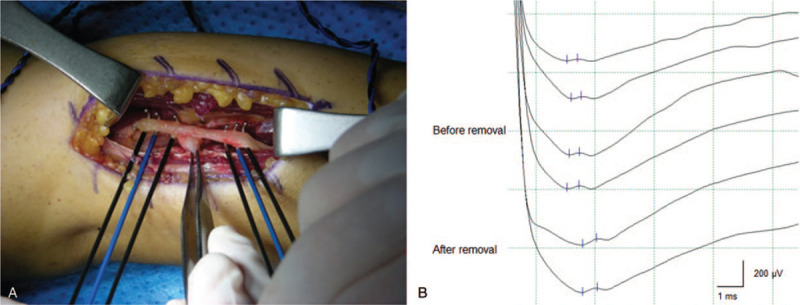
Nerve action potentials recorded on the posterior interosseous nerve (PIN) before and after ganglion cyst removal (B). J-shaped triple electrodes were applied on the PIN for both stimulation and recording (A).

For NAP recording, J-shaped triple electrodes were used for both stimulation and recording. The stimulation intensity was set to 1 mA, and the duration was 0.05 ms with a square pulse. The distance between the two electrodes was 4 cm. For stimulation and recording, the nerve was elevated and contact with fluid or adjacent soft tissue was avoided with minimal tension (Fig. 5A). The sweep speed was 1 ms/div, and the recording sensitivity was initially set at 200 uV/div. The filter setting was 5 − 3000 Hz.^[6,9]^ In our case, in the NAP study conducted before the ganglion cyst removal, NAP was stably evoked. Therefore, neuronal connectivity was confirmed. In addition, NAP was stably evoked after ganglion cyst removal (Fig. 5B).

**Figure 6:**
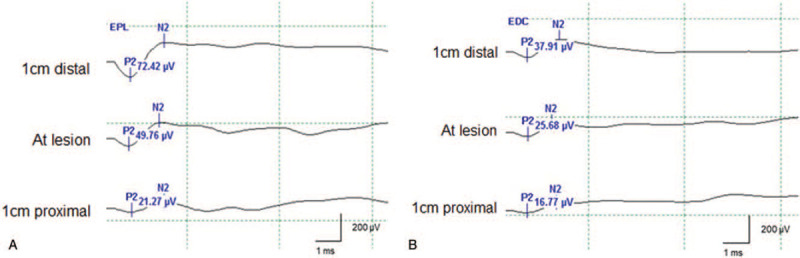
Compound motor action potentials (CMAP) of the extensor pollicis longus (EPL) (A) and extensor digitorum communis (EDC) (B) muscles obtained 1 cm proximal, at the lesion, and 1 cm distal to the point of the ganglion cyst. Both EPL and EDC showed a decrease in CMAP amplitude across the ganglion cyst.

For the inching study, a monopolar ball tip probe was used as a stimulation electrode, and the recording electrodes were applied to the EPL and EDC muscles using subdermal needle electrodes. The stimulation protocol was the same as the NAP. The inching test was performed three times: 1 cm proximal, at the lesion, and 1 cm distal to the location of the ganglion cyst. In our case, both EPL and EDC muscles showed a decrease in CMAP amplitudes across the cystic lesion, confirming a neural insult derived from the compression of the ganglion cyst. In addition, as CMAP was induced at the proximal site of the ganglion cyst, neuronal continuity was also confirmed (Fig. 6).

Total intravenous anesthesia was used for surgery. A single bolus of neuromuscular blockade was applied before intubation, and no additional muscle relaxant was applied during the surgery. To exclude the effect of the low temperature on nerve conduction, we routinely administered warm saline irrigation before each recording of NAP and CMAP.

### 2.4 Outcomes

The patient's serial muscle power changes and electrodiagnostic findings are summarized in Table 1, and were double-checked by a surgeon and a physiatrist. At the time of discharge, the patient showed greater wrist extensor muscle strength than before surgery, with a mMRC grade of 3+. However, there was no change in the muscle power of the finger and thumb extensors. After discharge, the patient regularly received physical treatment until 3 months after the surgery.

**Table 1:** Serial evaluations of the patient's muscle power and electrodiagnostic results.

		Time of evaluation			
Examination	Muscle	Preoperative	Postoperative	1 mo	3 mo
Manual muscle test (Modified MRC)	Wrist Ext	2+	3+	4	4
	Finger Ext	1	1	2	3
	Thumb Ext	1	1	2-	2
CMAP amplitude (mV)	EDC	2.1	–	1.6	2.2
	EIP	2.9	–	3	2.8
Electromyography	EDC	DP (3+), PIP	–	DP (2+), PIP	DP (1+), RP, CIP
	APL	DP (3+), SIP	–	DP (2+), SIP	DP (1+), RP, PIP
	EPL	DP (3+), SIP	–	DP (3+), SIP	DP (2+), RP, SIP
	EIP	DP (3+), SIP	–	DP (2+), PIP	DP (1+), RP, PIP

APL = abductor pollicis longus, CIP = complete interference pattern, CMAP = compound motor unit action potential, DP = denervation potentials, EDC = extensor digitorum communis, EIP = extensor indicis proprius, EPL = extensor pollicis longus, Ext = extensor, MRC = Medical Research Council scale, PIP = partial interference pattern, RP = reinnervation potentials, SIP = single interference pattern.

In the evaluations performed 1 month after the surgery, the patient's mMRC grade in the wrist extensor improved from 3+ to 4. Meanwhile, the mMRC grades of both her finger and thumb extensors was 2, showing no significant change. The NCS showed no significant change in the right EDC and EIP CMAP amplitudes, which were still less than 50% of those of the left side. On EMG, the degree of denervation potentials was decreased in the EDC, APL, EPB, and EIP muscles. However, reinnervation potentials were not observed. The interference pattern in the EIP was slightly improved.

In her 3-month postoperative visit, the patient's mMRC grade in her finger extensor had improved from 2 to 3. However, her thumb extensor was still weak, with an mMRC grade of 2. There was no obvious change in the CMAP amplitude on the NCS. On EMG, the degree of denervation potentials in the EDC, APL, EPB, and EIP muscles continuously decreased. The muscles also presented polyphasic, long duration, and large-amplitude motor unit action potentials, which provided evidence of neural reinnervation. The interference patterns in the EDC and APL muscles were also improved.

## 3 Discussion

In peripheral nerve surgery, the application of IONM makes it possible to immediately and reliably confirm the degree of nerve injury.^[10]^ This means that IONM can be used for purposes other than its main one, namely the monitoring of adverse neural events during surgery.^[11]^ Therefore, we suggest through our case presentation that IONM can be used as a tool to predict postoperative prognosis as well as to support decision-making in the surgical process by providing neurophysiological information on neural connectivity, whose assessment has previously been limited to visual inspection.^[6,12]^ In addition to IONM, preoperative electrodiagnostic tests can improve the assessment of the degree of nerve damage.^[13]^ According to Sunderland's classification,^[14]^ a type 1 injury does not require surgical treatment. However, in patients with a higher degree of neural injury, surgical exploration should be considered. In particular, in the case of type 2, 3, and 4 injury, Wallerian degeneration occurs while neural continuity is maintained. Therefore, if necessary, surgical decompression should be considered at the appropriate time, and better prognosis can be expected through decompression.^[8]^ On the contrary, if no response to NAP or CMAP is observed, the injury can be considered type 5. In this case, the surgeon should consider primary repair, including nerve grafts.^[15,16]^ Therefore, if preoperative electrodiagnostic test and IONM are combined, an accurate approach to the functional integrity of the damaged nerve is possible, which is helpful in determining the operation and its surgical method.

In our case, weakness persisted for more than 2 months, and there was no recovery of motor function during that period. However, we confirmed on the preoperative electrodiagnostic test that axonal continuity remained. In addition, since the localization of the lesion was possible through additional imaging tests, the surgical treatment could be determined. On IONM, NAPs were induced both before and after the removal of the ganglion cyst. Although the CMAP amplitude decreased when the proximal side of the ganglion cyst was stimulated in the inching test, it was still induced. Thus, we were able to confirm the functional connectivity of the compressed PIN within the surgical field.

In a previous study, when NAP was induced across the lesion, improvement of the mMRC to a grade 3 or higher was achieved in 90% or more of the cases. However, in cases where NAP was not induced, only 56% showed improvement to grade 3 or higher.^[7]^ Therefore, positive NAP responses across the lesion predict good prognosis. In addition, such responses can be seen as a factor that can predict the recovery of motor functions through decompressive surgery. In our case, the EMG performed 3 months after the surgery revealed reinnervation via collateral sprouting in all examined muscles, which is likely to lead to neuronal regeneration in the future.^[17]^

In the neurological evaluation conducted 3 months after the surgery, even though the patient's finger and thumb extensors showed some improvement in muscle strength, such improvement was not sufficient for securing functional recovery, a fact that could be explained by the following reasons. Neural reinnervation takes time: For collateral sprouting, it takes about 2–5 months.^[18]^ Meanwhile, nerve regeneration, the main mechanism of neural reinnervation, proceeds at a rate of 1.5 − 2.0 mm/d, and the growth rate decreases as regeneration proceeds in the distal direction.^[19]^ It is also highly probable that the delay of surgery while performing conservative treatment for 3 months reduced the recovery potential. In addition, since the patient had already progressed with the atrophy of PIN-innervated muscles at the initial evaluation in our hospital, even if neurological regeneration occurred, muscle recovery might be limited.^[20]^ Had the ganglion cyst on the PIN been confirmed through imaging studies within a short time after the onset of symptoms, followed by early surgery, the patient would have had a better prognosis. This suggests that imaging studies such as MRI and ultrasound should be actively considered in addition to conventional electrodiagnosis in patients with suspected compressive peripheral neuropathy. In addition, if there is no recovery from conservative treatment, active surgical repair even within 3 months will help preserve the undamaged nerve fascicles as much as possible.^[7]^

In conclusion, we report a case of surgical use of IONM in a patient with PIN entrapment due to a ganglion cyst. Preoperative electrodiagnosis and IONM confirmed neuronal connectivity and functional integrity. In peripheral nerve surgery, the role of IONM is to provide the basis for surgical decisions and to predict the patient's prognosis.

## Author contributions

**Conceptualization:** Dougho Park, Dong Young Kim, Seung Bum Chae.

**Investigation:** Dougho Park, Dong Young Kim, Yoon Sik Eom.

**Methodology:** Dougho Park, Dong Young Kim.

**Supervision:** Yoon Sik Eom, Sang-Eok Lee, Seung Bum Chae.

**Writing – original draft:** Dougho Park, Dong Young Kim.

**Writing – review & editing:** Sang-Eok Lee, Seung Bum Chae.
